# Neuronal-Glial Interactions Maintain Chronic Neuropathic Pain after Spinal Cord Injury

**DOI:** 10.1155/2017/2480689

**Published:** 2017-08-29

**Authors:** Young S. Gwak, Claire E. Hulsebosch, Joong Woo Leem

**Affiliations:** ^1^Department of Physiology, College of Korean Medicine, Daegu Haany University, Daegu, Republic of Korea; ^2^Department of Neurobiology and Anatomy, The University of Texas Health Science Center at Houston, Houston, TX 77030, USA; ^3^Department of Physiology, Yonsei University College of Medicine, Seoul, Republic of Korea

## Abstract

The hyperactive state of sensory neurons in the spinal cord enhances pain transmission. Spinal glial cells have also been implicated in enhanced excitability of spinal dorsal horn neurons, resulting in pain amplification and distortions. Traumatic injuries of the neural system such as spinal cord injury (SCI) induce neuronal hyperactivity and glial activation, causing maladaptive synaptic plasticity in the spinal cord. Recent studies demonstrate that SCI causes persistent glial activation with concomitant neuronal hyperactivity, thus providing the substrate for central neuropathic pain. Hyperactive sensory neurons and activated glial cells increase intracellular and extracellular glutamate, neuropeptides, adenosine triphosphates, proinflammatory cytokines, and reactive oxygen species concentrations, all of which enhance pain transmission. In addition, hyperactive sensory neurons and glial cells overexpress receptors and ion channels that maintain this enhanced pain transmission. Therefore, post-SCI neuronal-glial interactions create maladaptive synaptic circuits and activate intracellular signaling events that permanently contribute to enhanced neuropathic pain. In this review, we describe how hyperactivity of sensory neurons contributes to the maintenance of chronic neuropathic pain via neuronal-glial interactions following SCI.

## 1. Introduction

Pain raises alertness and prevents potential or actual damage to the human body. Neurotrauma to the central nervous system (CNS), such as spinal cord injury (SCI), causes neuropathic pain throughout the entire body; in contrast, peripheral nerve injury (PNI), such as sciatic or spinal nerve ligation, causes regional neuropathic pain [1]. In somatosensory system, both SCI and PNI eventually alter the transmission of ascending somatosensory modality that originates from skin tactile and thermal receptors, such that sensory neurons conduct altered discrimination of sensory inputs from the periphery to the higher brain regions, including the brain stem, thalamus, and cortex [1, 2]. Consequently, altered sensory neural pathways respond to nonpainful stimuli as painful stimuli (a phenomenon called allodynia) and enhance the pain in painful stimulation (a phenomenon called hyperalgesia) compared to the normal sensitivity [1]. These enhanced neuronal response properties to stimuli, or neuronal hyperactivity, which manifest pain hypersensitivity, suggest that once neurons lose their ability to accurately encode the somatosensation, they are more sensitive and easier to activate [3, 4]. Thus, neuronal hyperactivity is a key factor in the development and maintenance of neuropathic pain following neurotrauma.

Over the last few decades, rodent animal models that mimic many aspects of human neuropathic pain symptoms have been developed. However, SCI-induced neuropathic pain has been more elusive to understand than PNI-induced neuropathic pain. The majority of studies of PNI-induced neuropathic pain have focused on ascending pain pathways from injured sites to the cortex without a regional mechanism, whereas studies of SCI-induced neuropathic pain have focused on ascending pain pathways with a regional mechanism, such as at-level, below-level, and above-level neuropathic pain, as well as glial activation [5]. There are several mechanisms that can account for neuronal hyperactivity in the CNS, which contributes to altered pain states. This outcome can be explained by a variety of nonexclusive mechanisms, including enhancement of spinal cord nociceptive processing by a decrease of the descending inhibition [6, 7], increases in concentrations of excitatory amino acids (EAAs) [8, 9], deafferentation hyperexcitability of spinal neurons and/or thalamic neurons [10, 11], increased efficacy of previously ineffective synapses [12, 13], and anatomical alterations in the spinal cord [14, 15]. Each of these may contribute to the mechanisms underlying persistent hyperactivity of spinal cord dorsal horn neurons [16, 17].

In the present review, we describe mainly neuronal-glial interactions and also briefly the activation of intracellular signaling pathway and reorganization of spinal synaptic circuits, which are important causes of dorsal horn neuronal hyperactivity leading to pain hypersensitivity following SCI.

## 2. Modeling Central Neuropathic Pain

Little attention has been given to mechanisms of chronic pain in SCI in the clinics, and it has only been in the last several years that animal models were developed to study the development and maintenance of central neuropathic pain-like behavior after SCI. The models include an intravascular photochemical reaction that occludes blood vessels, producing spinal cord ischemia with subsequent trunk mechanical allodynia [18, 19]; anterolateral lesions of the spinal cord in monkeys and rats that produce overgrooming and mechanical allodynia [20, 21]; a clip compression model in which the thoracic spinal cord is compressed by 35 g or 50 g clip demonstrates mechanical hyperalgesia in the hindlimbs [22]; quisqualic acid (an AMPA/kainate and a group I glutamate metabotropic receptor agonist) injection into the dorsal horn produces overgrooming [23]; and a spinal hemisection [24, 25] and spinal contusion models [26–28] demonstrate mechanical allodynia in both the hindlimbs and forelimbs. The spinal contusion model best parallels the injury profile described in patients with SCI [29].

**Figure 1:**
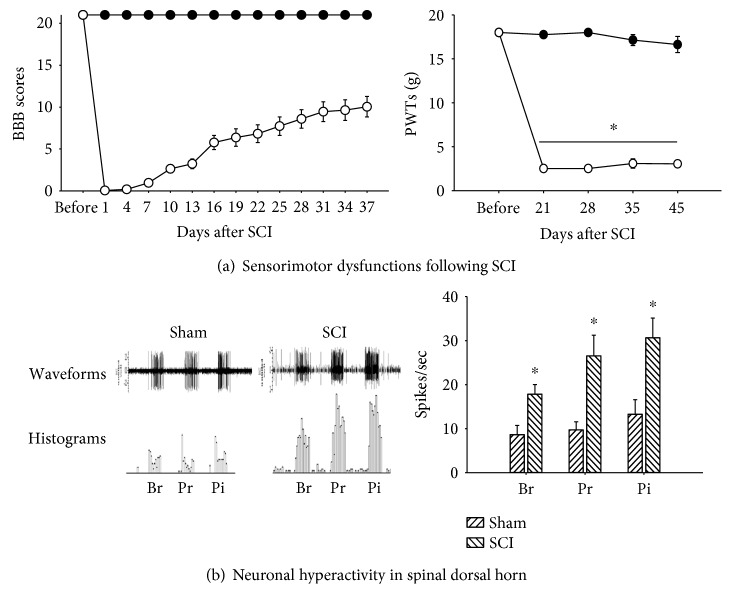
Neuropathic pain behavior and neuronal hyperactivity following SCI. ((a), left) Compared to the sham group (closed circle), the SCI group (open circle) shows complete loss of locomotion at early phase (days 1 to 4) following contusive spinal cord injury (SCI) at the thoracic segment T10. However, three weeks after SCI, animals gradually recover locomotion, showing BBB scores over 7, and are enable to position and withdrawal responses. The BBB scores are averaged by left and right sides of the hindlimbs. ((a), right) Pain behaviors were measured by paw withdrawal thresholds (PTWs), which are determined by quantitative assessment of withdrawal behaviors ([33]). On three weeks after SCI, pain behaviors develop (decrease of PWTs scores) and maintained. (b) On four to five weeks after SCI, lumbar spinal wide dynamic range (WDR) dorsal horn neurons (neurons that respond to all three stimuli: brush, pressure, and pinch in their peripheral receptive field) display significantly increased evoked activity in response to all three stimuli (10 seconds each) compared to the sham group (modified from Gwak et al. [28]). Br: brush, Pr: pressure, and Pi: pinch stimulation. ^∗^*p* < 0.05 versus the sham group.

It is noted that the adequate evaluation of animal pain behaviors after SCI should be considered by sensorimotor activity. Because SCI disrupts ascending sensory and descending motor pathways, the activity of sensorimotor is critical to the evaluation of animal pain behaviors. In motor studies following SCI, we and others assess changes in locomotion using the BBB open-field test scale [30, 31] to demonstrate that the hindpaws are plantar placed and weight bearing and consequently that the animal can position and withdraw its hindpaw in response to somatosensory stimuli; and measure exploratory activity [8, 9] as well as the number of ultrasonic vocalizations during rest as measures of spontaneous behaviors that are consistent with a noxious experience [32]. In sensory studies following SCI, the sensitivity to mechanical, thermal, and chemical stimulation determines the painful sensation. Because painful stimuli trigger avoid and withdrawal behaviors, the evaluation of pain behavior can be determined by the number of withdrawals or the threshold values for the withdrawals [33]. The increased number of withdrawal and decreased thresholds correlate well with dorsal horn neuronal hyperactivity. In fact, when animals with SCI are submitted to tactile stimulation of the hindpaw, decreased threshold for hindpaw withdrawals occurs concomitant with increased discharge rate of dorsal horn sensory neurons (Figure 1). In addition, the increase of receptor/ion channel expression in the CNS following SCI shows a clear correlation with neuropathic pain behaviors [8, 34, 35].

In studies of SCI patients with or without spontaneous and stimulus-evoked pain, the predominant mechanism underlying SCI-induced neuropathic pain involves neuronal hyperactivity at the levels of the spinal cord and brain [36–41]. For example, in SCI patients, the blockade of peripheral inputs by lesioning of the dorsal root entry zone (DREZ) of the spinal cord and by pharmacological inhibition of spinal NMDA receptors or activation of peripheral opioid receptors effectively attenuates the increased response of central neurons to repeated C-fiber stimulation as well as pain hypersensitivity [42–44]. In addition, SCI patients show the higher concentrations of proinflammatory cytokines, such as interleukin 1 (IL-1), IL-6, and tumor necrosis factor alpha (TNF*α*), in the serum and the microglial activation in the injured spinal cord [45, 46]. The elevated serum levels of cytokines and the activation of spinal microglia are also reported from studies in rodent SCI models [47–52]. Moreover, the clinical findings in SCI patients, especially regarding neuronal hyperactivity, are also observed in rodent SCI models (see below). Thus, animal models of SCI can serve as an important source to understand the mechanisms of neuropathic pain in SCI patients.

## 3. Hyperactivity of Spinal Sensory Neurons after SCI

Neuronal hyperactivity is defined as enhanced spontaneous excitability of neurons or abnormally increased neuronal activity in response to mechanical, thermal, and chemical stimulation. Neuronal hyperactivity has been well documented in electrophysiological studies in rodent SCI models and is characterized by increased evoked and spontaneous action potential frequencies, lowered thresholds for action potential generation, and prolonged afterdischarge activity [52–56]. Thus, hyperactivity of sensory neurons mediates enhanced nociceptive processing in pathological pain states. In addition, electrophysiological studies have demonstrated that SCI causes altered neuronal response properties of the at-level (near the injured site) and below-level (several segments caudally remote from the injured site) spinal dorsal horn regions as well as the supraspinal regions, specifically the gracile nucleus and the thalamic ventral posteriolateral (VPL) and posterior thalamic (PO) nuclei [57–60]. Cumulatively, these data suggest that SCI produces neuronal hyperactivity along somatosensory pathways in the CNS.

SCI causes direct (primary) and indirect (secondary) damages to spinal dorsal horn sensory neurons. According to their electrophysiological properties, spinal sensory neurons are classified into three types. Low-threshold (LT) neurons show good responses to weak stimuli, such as vibration, touch, and brushing at the peripheral receptive field. In contrast, these neurons do not respond to strong stimuli such as pinching. High-threshold (HT) neurons show good responses to both moderate and strong stimuli. Wide dynamic range (WDR) neurons respond well to both weak and strong stimuli, so that WDR neuronal response increases gradually as the stimulation intensity increases [61–63]. *In vivo* extracellular recording is a useful tool for examining neuronal response properties to mechanical stimulation. Using this technique, we and others have reported that three types of spinal sensory neurons have significantly altered responses to various mechanical stimuli after SCI. Specifically, all neuronal types show significantly increased firing activity after SCI compared to normal in response to stimulation applied at the peripheral receptive field [54, 55]. Moreover, the enhanced responses to peripheral stimuli are sustained in rodent SCI models over many months and may persist permanently [5]. More importantly, SCI induces phenotypical changes in spinal sensory neurons. The proportions of dorsal horn neurons with WDR characteristics were increased, whereas the proportions of HT and LT neurons were decreased. However, altered electrophysiological properties of dorsal sensory neurons are not the only factor causing neuronal hypersensitivity; anatomical changes followed by alteration of synaptic circuits are also an important factor for hyperactivity of spinal sensory neurons (see below).

### 3.1. Reorganization of Synaptic Circuits after SCI

Over the last few decades, morphological studies have suggested that the maintenance of neuronal hyperactivity depends on maladaptive synaptic circuits. For example, traumatic SCI essentially destroys sensory-mediating afferent pathways, thereby resulting in the reorganization of synaptic circuits induced by neuronal cell death, degeneration, or primary afferent axon expansion [64, 65]. Although the spinal nervous system has compensatory and neuroprotective mechanisms for recovery, it is well documented that neuronal-glial interactions impedes these mechanisms. For example, endogenous nerve growth factor (NGF) released by activated microglia, a subset of astrocytes and other inflammatory cells, facilitates maladaptive compensatory events, such as regeneration or sprouting of primary afferent fibers, at regions near the injured site and at remote regions [66–68]. Thus, at the single-cell level, incoming primary afferent signals can be amplified in the dorsal horn due to increases of projection pattern and synaptic input [25, 68]. In addition, SCI has been shown to cause dendritic spine dysgenesis in the spinal dorsal horn, thus contributing to neuropathic pain via activation of Rac1, a small signaling G-protein [69]. Activated glia, however, have also been shown to release growth inhibition factors (e.g., NG-2, neurocan, and brevican) within a few days, thereby preventing compensatory axonal regeneration and regrowth [70–73]. Another study concludes that activated glia contribute to the reorganization of synaptic circuits at the spinal dorsal horn and supraspinal regions including the VPL thalamic nucleus [74, 75]. In a recent study, Lee-Kubli et al. report that SCI caused astrocytic and microglial activation in the spinal cord and satellite glial cell activation in DRGs, respectively, and suggest that SCI-induced neuronal-glial interactions may occur throughout the entire nervous system [76].

It is well known that maladaptive synaptic reorganization induced by activated glial cells contributes to the glial-neuronal interactions at the synapse at the so-called tripartite synapse following traumatic CNS injury [77]. For example, the extension of microglial and astrocytic processes into and near synaptic clefts following CNS injury allows alterations to thousands of synaptic clefts and vastly altered neural networks. These tripartite clefts (presynaptic and postsynaptic neuronal structures with contributions from activated microglia and/or activated astrocytes in the microenvironment) facilitate transmission of pain-mediating substances produced by activate glial cells, such as TNF*α*, BNDF, interleukins, and ROS [78, 79]. Therefore, posttraumatic neuronal and glial mechanisms contribute to the reorganization of synaptic circuits throughout the entire nervous system and can result in chronic central neuropathic pain throughout the entire body [4, 75].

### 3.2. Intracellular Signaling Cascades after SCI

**Figure 2:**
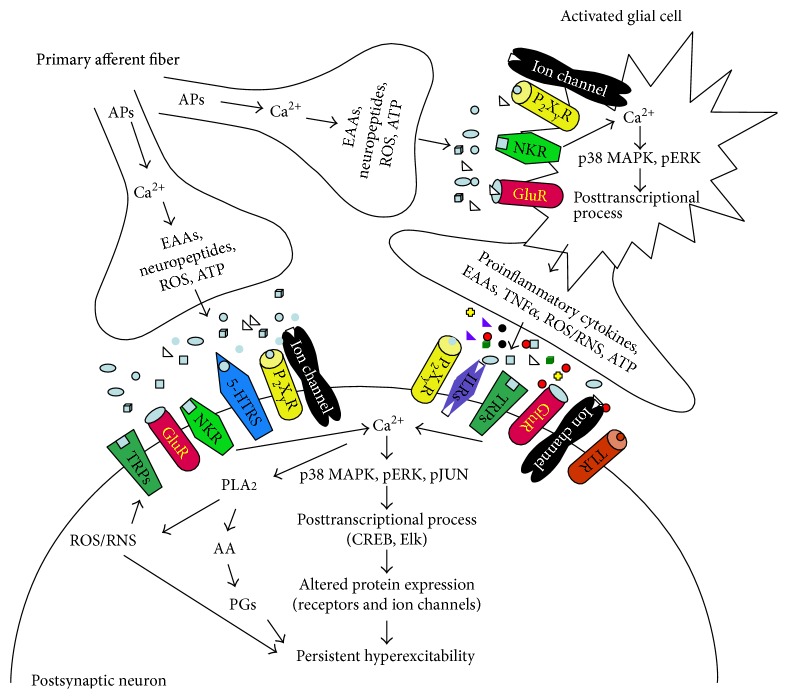
Intercellular and intracellular mechanisms driving persistent neuronal hyperactivity following SCI. After SCI, activated primary afferent fibers release pain-mediating substances on both postsynaptic neurons and activated glial cells. Elevation of calcium ion concentrations in neurons and activated glial cells triggers similar intracellular downstream events in both neurons and glial cells, that is, activation (phosphorylation) of p38-MAPK and ERK, followed by activation of posttranscriptional and posttranslational processes that result in altered protein and ion channel expression. In neurons, elevated calcium ion concentrations also trigger activation of calcium-dependent (direct) pathways and calcium-independent (indirect) PLA_2_ pathways, followed by increased AA, ROS, and PG synthesis. These effects contribute to the development of persistent neuronal hyperactivity. Activated glial cells release gliotransmitters to the extracellular space, thereby activating receptors and/or ion channels in the neuronal membrane. Subsequently, gliotransmission activates neural membrane receptors and/or ion channels, thereby triggering a massive influx of cations (Na^+^ and Ca^2+^) into the intracellular compartments of neurons. This positive feedforward cycle maintains persistent neuronal hyperactivity, which plays a key role in the development of neuropathic pain after SCI. p38-MAPK: p38 mitogen-activated protein kinases; ERK: extracellular signal-regulated kinases; 5-HTRs: 5-serotonin receptor; AA: arachidonic acid; APs: action potentials; EAAs: excitatory amino acids; ILRs: interleukin receptors; NKR: neurokinin receptor; PGs: prostaglandins; PLA_2_: phospholipase A_2_; ROS/RNS: reactive oxygen/nitrogen species; TLRs: toll-like receptors; TRPs: transient receptor potential channels.

In spinal dorsal horn sensory neurons, the predominant events immediately after SCI are production of intense discharge activity from injured and adjacent axons, dramatic increased extracellular concentrations of glutamate and proinflammatory cytokines, and increased reactive oxygen species (ROS) production, followed by activation of various protein kinases and other enzymes [80, 81]. Thus, immediate electrical and neurochemical events post-SCI activate pain-mediating substance transmission in synaptic clefts between neurons and glial cells (see Figure 2). Consequently, the activation of membrane-bound receptors and ion channels triggers a massive influx of cations into intracellular compartments that is followed by activation of intracellular biochemical events, thereby triggering activation of transduction and translation cascades.

It is well documented that activation of intracellular downstream pathways triggers consistent hyperactivity of dorsal horn sensory neurons following SCI [82, 83]. The increase of intracellular calcium ions induced by activation of NMDA receptors and voltage-dependent calcium channels triggers intracellular downstream pathways. For example, calcium ions facilitate activation of the protein kinase A (PKA), protein kinase C (PKC), and calcium-calmodulin-dependent kinase II (CaMKII) pathways. Simultaneously, activation of MAPK and extracellular signal-regulated kinase (ERK) initiates activation of transcription factors such as NF*κ*B, ELK, and CREB, which results in altered gene expression. Transduction and translation cascades can also contribute to persistent sensory neuronal hypersensitivity. Additionally, activation of glutamate metabotropic receptors and neurotrophin receptors, such as Trk, also induces activation of PKC ➔ MEK ➔ MAPK pathways [83, 84]. Recently, more direct evidence indicates that MAPK family-CREB pathways are actively involved in the dorsal horn sensory neuronal hyperactivity and central neuropathic pain following SCI. Specifically, SCI results in activation of the p-38 MAPK-phosphorylated CREB (pCREB) pathway, which modulates sensory neuronal hyperactivity and central neuropathic pain after SCI. The reduction in the phosphorylated form of MAPK, p-38 MAPK, is also shown to attenuate the neuropathic pain behavior and sensory neuronal hyperactivity following SCI [55]. SCI-induced upregulation of pCREB expression persists over a month in dorsal horn sensory neurons after SCI and is attenuated by MAPK inhibition. Thus, protein kinase transcriptional factor pathways alter target gene expression, thereby driving phosphorylation of multiple receptors and ion channels. These effects contribute to the persistent hyperexcitability state of dorsal sensory neurons [85]. Therefore, downstream and upstream intracellular cyclical cascades contribute significantly to persistent neuronal hyperactivity that leads to chronic central neuropathic pain following SCI.

Other intracellular pathways also play a role. For instance, high concentrations of intracellular calcium ions trigger activation of phospholipase A_2_ (PLA_2_). PLA_2_ hydrolyzes the cell membrane and produces free fatty acids, thereby producing lipid metabolites. Specifically, calcium-dependent cytosolic PLA_2_ (cPLA_2_) and secretory PLA_2_ (PLA_2_) break down phospholipids and produce arachidonic acid and other lipid metabolites such as prostaglandins and leukotrienes. Interestingly, these lipid metabolites are strong candidates for pain development and maintenance [86–88]. In addition, active calcium-independent PLA_2_ (iPLA_2_) is a powerful mediator of the production of free radicals and pain inducers, such as reactive oxygen species (ROS), reactive nitrogen species (RNS), and MAPK family downstream pathways [89, 90]. Therefore, PLA_2_-mediated lipid membrane hydrolysis causes activation of intracellular events that result in membrane-bound receptor and ion channel dysfunction [91]. In particular, ROS trigger the release of glutamate via the TRPV1 and TRPA1 channels [92, 93] and production of proinflammatory cytokines via microglial NADPH oxidase 2 (NOX2) pathways [94], making sensory neurons easier to activate. Recently, Kiyoyuki et al. report that the lipid metabolite leukotriene facilitates NMDA-inward current via intracellular G-proteins and neuronal hyperactivity in the spinal dorsal horn [95]. These reports suggest that ROS-mediated lipid metabolites activate membrane-bound channels or receptors that contribute to sensory neuronal hyperactivity and chronic neuropathic pain following SCI. In addition, we previously proposed that activation of iPLA_2_ contributes to neuropathic pain following SCI. For example, SCI resulted in the upregulation of iPLA_2_ in spinal dorsal horn sensory neurons and using *in vivo* extracellular recordings, we showed that the administration of an iPLA_2_ inhibitor attenuated neuronal hyperactivity [96]. Another study demonstrated that lipid metabolites such as arachidonic acid-containing phosphatidylcholine (AA-PC) contribute to neuropathic pain and increase the levels of reactive microglia/astrocytes in the spinal dorsal horn, suggesting that regulation of phospholipids is an important factor for neuropathic pain [97]. Therefore, these data suggest that lipid metabolites contribute to the maintenance of sensory neuronal hyperactivity following SCI.

Taken together, these studies indicate that SCI produces long-lasting or persistent hyperactivity of spinal dorsal horn sensory neurons via altered intracellular signaling pathways. In particular, activation of PLA_2_-ROS pathways perpetuate receptor/ion channel activation and membrane property changes via transcriptional/translational events that modulate the expression of specific genes and proteins, making glutamate receptor-bearing neuronal membranes hyperexcitable after SCI.

## 4. The Role of Glial Cells in SCI-Induced Neuropathic Pain

In the CNS, glial cells are composed of astrocytes, microglia, and oligodendrocytes; in the peripheral nervous system, oligodendrocytes are replaced by Schwann cells. Glial cells are intimately associated with neurons and their processes form complex wrapping patterns around nerve processes during development. Glial cells actively contribute to the maintenance of ionic balance and other regulatory processes that enable homeostasis under physiological conditions in the nervous system [98, 99]. Glial cells are easily activated by injury, stress, and inflammation. Activated glial cells are key cellular contributors in the development of abnormal physiological roles that result in maladaptive synaptic circuits. Subsequent alterations in neuronal-glial circuits have been shown to contribute to the enhancement of pain transmission [100].

Throughout the CNS, it is well established that SCI results in astrocytic and microglial activation. Using a strict anatomical or morphological definition, astrocytic and microglial activation is “somatic hypertrophy and thickened branches.” It is well documented that SCI produces astrocytic and microglial somatic hypertrophy and thickened branches compared to the resting state [101, 102]. Previously, we have proposed that temporal and spatial astrocytic and microglial activation occurs after SCI. In addition, activated astrocytes and microglia in both superficial and deep dorsal horn laminae contribute to persistent activation of dorsal horn neurons for several months following SCI [5]. However, the mechanisms contributing to the development of hypertrophy and altered function in activated astrocytic and microglial cells are not yet precisely understood, although a variety of possible mechanisms have been proposed. First, astrocytic and microglial cell membranes are more resistant to somatic volume changes than neuronal cell membranes. This property results in astrocytic and microglial cell hypertrophy rather than death after neural trauma [103]. Secondly, potassium homeostasis regulates glial cell volume [104]. Specifically, SCI produces a massive influx and accumulation of glutamate (through high affinity glutamate transporters) into glial cells, followed by uptake of K^+^ (by Na^+^/K^+^-ATPase) ions. High intracellular concentrations of K^+^ in glial cells trigger energy-independent influxes of Cl^−^, K^+^, and HCO_3_^−^ from extracellular to intracellular compartments. Subsequently, activation of anion channels triggers H_2_O accumulation, which produces somatic hypertrophy [105]. Thirdly, activation of the Na^+^/K^+^/Cl^−^ cotransporter (NKCC) results in glial cell hypertrophy. For example, SCI-induced overproduction of ROS increases NKCC activity and induces an influx of Na^+^ ions that results in hypertrophy of activated glial cells [106]. However, the morphological changes of astrocytic and microglial cells do not fully account for the role of activated glial cells in pain transmission following SCI. A recent report suggests the roles of oligodendrocytes in the neuropathic pain. For example, IL-33, a member of the IL-1 family of cytokines, expressed in oligodendrocytes within the spinal cord after peripheral nerve injury in mice involves the generation of neuropathic pain behavior, and this pain behavior is attenuated by inhibition of IL-33/ST2 (IL-33 receptor) signaling [107]. We have previously reported that an active form of one intracellular signaling kinase, pCaMKII, is upregulated in oligodendrocytes in the dorsal column of the spinal cord in animals showing neuropathic pain behavior after SCI, and SCI-induced pain behavior is reduced by preventing CaMKII activation [108]. It is also demonstrated that genetic ablation of oligodendrocytes in mice, which causes axonal pathology in the spinal dorsal horn and spinothalamic tracts, leads to the development of neuropathic pain behavior [109].

Another reasonable hypothesis to explain glial activation involves the functional contribution of these cells to neurodegeneration. Activated glial cells induced by SCI increase the release of neurotransmitters, proinflammatory cytokines, ROS/RNS, ATP, and nitric oxide (NO) [110–113]. These substances are actively involved in pathophysiological conditions such as apoptosis, Parkinson's disease, Alzheimer's disease, and neurodegeneration [114–116]. For example, inhibition of iNOS (inducible nitric oxide synthase) and glial activation reduce neuronal apoptosis and promote the neuronal functions [117]. They are also powerful mediators of enhanced pain transmission following SCI. These pain-mediating substances facilitate activation of neuronal membrane-bound receptors and ion channels. Activation of cationic channels initiates calcium influx and activates intracellular cascades, followed by the activation of transcription factors in both presynaptic and postsynaptic neurons. We hypothesize that the abnormal altered biochemical pathways as a result of glial activation causally contribute to the maintenance of hyperactivity of spinal sensory neurons after SCI [118, 119].

In the past decade, several studies have reported that peripheral nerve injury-induced glial activation robustly contributes to peripheral neuropathic pain [120, 121]. In addition, some studies have presented data to support the idea that persistent spinal astrocytic and microglial activation contributes to mechanical allodynia and neuronal hyperactivity following SCI [122, 123]. There are several ways in which glial cells participate in SCI-induced neuropathic pain. First, proinflammatory cytokines released from activated glial cells after SCI cause hyperactivity of dorsal horn sensory neurons to result in neuropathic pain. Detloff et al. demonstrate that TNF*α* and IL-1*β* contribute to the development of mechanical allodynia after SCI, whereas IL-6 contributes to the maintenance of mechanical allodynia [124]. In addition, SCI causes activation of trkB.T1- (a truncated isoform of the BDNF receptor) mediating signaling in astrocytes, resulting in the increase of astrocyte proliferation and mechanical allodynia. However, the trkB.T1 KO mice show decreased astrocyte proliferation and reduced mechanical allodynia [125]. Second, activated glial cells by SCI modulate inhibitory tone within the spinal cord, causing increased excitability of dorsal horn sensory neurons to lead to neuropathic pain. It has been shown that SCI decreases inhibitory GABAergic function and increases spinal WDR neuronal activity in the spinal dorsal horn laminae II–V [126]. In addition, inhibition of glial activation prevents downregulation of glutamic acid decarboxylase (GAD)_65_, the GABA synthase enzyme, in spinal sensory neurons [4]. Third, decreased expression of glutamate transporter (GLT1) in glial cells after SCI causes hyperactivity of dorsal horn sensory neurons to lead to subsequent neuropathic pain. Indeed, local downregulation of GLT1 at the neuronal-astrocytic synaptic cleft is shown to facilitate glutamate-mediated transcriptional cascades, thereby resulting in increased responses of dorsal horn sensory neurons to tactile and thermal stimulation of the peripheral receptive field [34]. Fourth, activated glial cells by SCI modulate intracellular signaling pathways to induce dorsal horn neuronal hyperactivity and pain hypersensitivity. In fact, it has been revealed that SCI-induced modulation of downstream elements including p-38 MAPK and ERK occurs in both neurons and glial cells [56, 85]. Multiple studies have demonstrated that pharmacological blockade of glial activation prevents neuronal and glial activation of p-38 MAPK and reduces hyperactivity of dorsal horn sensory neurons to tactile and thermal stimuli, as well as attenuating mechanical allodynic behavior at both “at-level” [56] and “below-level” [127] regions following SCI.

Interestingly, the spatial distributions and localizations of activated glial cells and activated MAPK family members show different patterns in different SCI animal models. In a hemisection lesion (dorsal to ventral) in the spinal cord, activation of p-38 MAPK is observed at neurons and microglia in the dorsal horn in remote caudal regions [127]. However, the hemisection SCI shows no p-38 MAPK activation in astrocytes, even though astrocytes are observed in the activated state, as demonstrated by morphological changes and increased levels of GFAP. In contrast, moderate/severe contusion SCI using the Infinite Horizon Impactor results in activation of p-38 MAPK and ERK in neurons, astrocytes, and microglia, as well as astrocytic and microglial activation in the at-level region [56]. However, contusion SCI using the NYU Impactor causes p-38 MAPK activation in microglia in caudal regions [122]. In addition, the induction of moderate contusion SCI using an OSU electromagnetic device results in activation of p-38 MAPK in neurons and microglial activation at caudal “below-level” regions rather than astrocytic activation [124]. Taken together, these data suggest that differential mechanical injuries of the spinal cord influence cellular and spatial distribution of p-38 MAPK activation in neurons and specific glial cell populations. However, intracellular downstream events share common pathways that result in the development and maintenance of neuropathic pain after SCI.

## 5. Neuronal-Glial Interactions in SCI-Induced Neuropathic Pain

Astrocytes and microglia express receptors and ion channels that are also expressed in neurons [128–130]. Consequently, neurotransmitters and neuropeptides released by primary afferent terminals initiate activation of membrane-bound receptors and ion channels at synaptic clefts between neurons and glial cells. Apart from neuronal activation (see above), SCI produces a long-lasting surge of extracellular glutamate [131] that is followed by significant increases in proinflammatory cytokine secretion, overexpression of membrane-bound receptors and ion channels, and altered expression of transporters in both neurons and activated glial cells [132–134]. Neuroanatomical and functional changes initiate glial cell activation, followed by increased release of gliotransmitters (transmitters released by glial cells in a process called “gliotransmission”), proinflammatory cytokines, ROS/RNS, and chemokines. These substances initiate activation of a positive feedforward cycle between neurons and glial cells that enables persistent neuronal hyperactivity.

**Figure 3:**
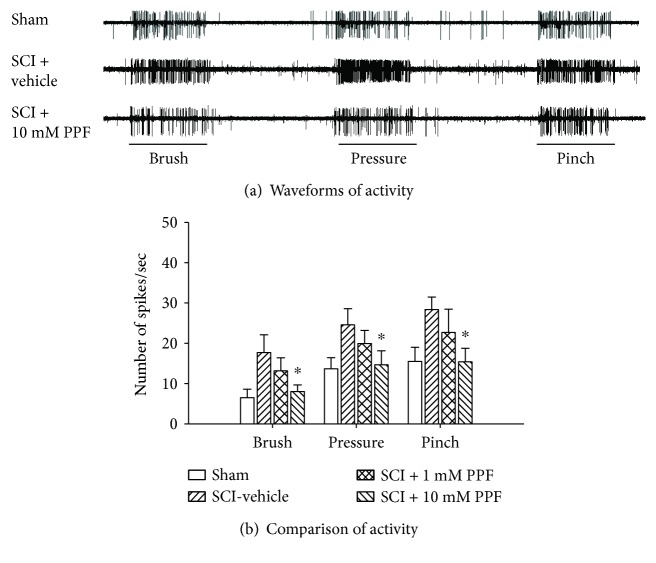
Attenuation of neuronal hyperactivity by spinal treatment with PPF following SCI in rats. (a) Typical spike activity during 10 sec (scale bar) of stimulation in the peripheral field during single neuron recordings from sham (top), SCI + vehicle (middle), and SCI + 10 mM PPF- (bottom) treated rats. (b) After SCI, lumbar spinal wide dynamic range (WDR) dorsal horn neurons displayed significantly increased evoked activity in response to peripheral stimuli (10 seconds each) in the SCI-vehicle group compared to the sham group. Two hours after spinal treatment with 10 mM PPF, this activity was significantly attenuated. In contrast, 1 mM PPF had no effect compared to the vehicle treatment (modified from Gwak et al. [4]). ^∗^*p* < 0.05 versus the SCI + vehicle group.

In *in vivo *electrophysiological studies, activated glia contribute significantly to sensory neuronal hyperactivity, as evidenced by wind-up, the frequency-dependent facilitation of C-fiber activation in spinal dorsal horn neurons [135]. For example, IL-1*β* treatment caused wind-up in spinal dorsal horn sensory neurons; moreover, this effect was attenuated by propentofylline (PPF), a glial modulator that inhibits astrocytic and microglial activation [136]. PPF is a therapeutic agent that acts by blocking the uptake of adenosine and inhibiting the production of phosphodiesterase (PDE). PPF inhibits GFAP (glial fibrillary acidic protein) production in astrocytes and OX-42 (CD11b) production in microglia/macrophages. Gwak and Hulsebosch have demonstrated that inhibition of glial activation induced by intrathecal PPF treatment attenuates sensitized WDR neurons in lumbar dorsal horn following low thoracic SCI ([123], Figure 3). In addition, peripheral neuropathic pain behavior is attenuated by PPF treatment [137, 138]. The pharmacological properties of PPF are thought to regulate the synthesis and release of proinflammatory cytokines [136], which are common substrates for pain transmission.

Proinflammatory cytokines enhance the expression of substance P, inducible nitric oxide synthase (iNOS), and cyclooxygenase (COX); changes that are followed by activation of p-38 MAPK and ERK [139], which initiate activation of transcription factors such as pCREB. Glial cells also participate in synaptic development in neurons. For example, upregulation of astocytic ephrin-B1 correlates with decreased vGlut1-positive glutamatergic input to CA1 neurons following traumatic brain injury. However, the ablation of astrocytic ephrin-B1 promotes the recovery of vGlut1-positive glutamatergic input to CA1 neurons [140]. Therefore, astrocytes make important contributions to synapse remodeling. In addition, more direct evidence that glial cells modulate neuronal excitability exists. For example, glial cells alter the expression of KCC2 and NKCC1, thereby decreasing GABAergic inhibitory tone and increasing glutamate release [141]. Other studies suggest that lipid metabolism-mediated glial activation contributes to neuropathic pain following SCI. For example, intrathecal treatment with lipid mediator lipoxin 44 (LXA4) prevents SCI-induced neuropathic pain and microglial activation [142]. These intracellular downstream and translational factor cascades modulate receptor expression and inflammatory cytokine production, thereby producing persistent hyperactivity of dorsal horn sensory neurons that contribute to the initiation and maintenance of central neuropathic pain following SCI [127].

Taken together, these morphological and functional alterations of activated glial cells contribute to multifactorial extracellular and intracellular signaling changes. These changes are followed by alterations in transmitter/receptor/transporter expression and activation states. Therefore, dysregulation of glial functions of activated glial cells can be termed a “gliopathy” [143] characterized by increased release of gliotransmitters, increased secretion of proinflammatory cytokines, upregulation of membrane-bound receptors/ion channels, and upregulation of transporters. In recent, Wu et al. report that SCI causes increase of cell cycle activation (CCA) at the injured spinal and thalamic levels. In addition, increased CCA significantly contributes to the neuronal hyperactivity and gliopathy. However, a systemic injection of flavopiridol (a pan-cyclin-dependent kinase inhibitor) reduces CCA, glial changes in the spinal dorsal horn, and neuropathic pain [144, 145]. Since normal glial-neuronal networks maintain homeostatic function of the nervous system, gliopathy may play a critical role in inducing the synaptic reorganization, synaptic changes in efficacy, alterations in neuronal excitability, and other maladaptive mechanisms that results in central neuropathic pain following SCI.

## 6. Clinical Applications and Limitations of Glial Activation

A number of animal studies have demonstrated that modulation of glial activation can have beneficial effects by attenuating neuropathic pain. However, the efficacy that can be achieved by this approach is still under debate. For example, intrathecal administration of the glial modulator PPF given intraperitoneally resulted in attenuation of SCI-induced astrocytic/microglial activation and neuropathic pain in animal models [4]. However, intrathecal PPF shows no significant effects on neuropathic pain induced by spinal nerve crush injury in animal models [146] and no decreased pain in postherpetic neuralgia patients [147], most likely due to differential responses of rodent and human glial cells. In addition, inhibition of microglial activation by an intraperitoneal injection of minocycline immediately after injury in the early phase attenuates both tactile and thermal hypersensitivity (neuropathic pain), but administration of minocycline 7 days after injury has no effects on neuropathic pain following peripheral nerve injury in animal models [148]. One recent study reports that neuropathic pain attenuation is not directly related to morphological changes of glial cells. They reported that oral administration of a p38 MAPK inhibitor attenuated neuropathic pain following SCI, without causing any morphological changes in astrocytes or microglia [149]. Currently, three glial-modulating agents are widely used in animal studies of neuropathic pain attenuation. Two of these agents, ibudilast (AV-411 and MN-166) and PPF, are inhibitors of phosphodiesterase (PDE); in contrast, minocycline is a synthetic tetracycline derivative. Since their ability to inhibit glial cell activation is reasonably correlated with their ability to attenuate neuropathic pain following SCI, these substances are strong candidates for attenuating neuropathic pain by inhibiting glial cell activation. However, after a number of clinical trials, the value of glial-modulating agents is still under debate. For example, minocycline treatment results in pain attenuation in the clinic [150], but PPF shows no pain attenuation in human trials [147]. Administration of ibudilast has been shown to attenuate peripheral nerve injury-induced and paclitaxel-induced pain in an animal study [151]; however, this effect is not observed in a clinical trial [152]. Although most animal studies have demonstrated that glial modulation is efficacious in treating neuropathic pain [153], future studies in humans must (1) characterize the effects of glial modulation on glial activation and pain severity and (2) optimize the administration route and application time in order to identify the most promising translational applications of glial modulation in human neuropathic pain [154].

## 7. Conclusion

Neuronal hyperactivity is a major factor in the development of chronic central neuropathic pain following SCI. While the development and maintenance of neuronal hyperactivity depends on neuronal events, glial activation also plays a key role through receptor activation and/or ion channel-mediated pathways. Therefore, neuronal-glial interactions initiate and maintain activation of membrane-bound proteins and subsequent intracellular downstream events. These events, in turn, result in persistent neuronal hyperactivity via positive feedforward cycles. In particular, persistent activation of astrocytes and microglia after SCI leads to morphologically and functionally altered glial cells in a “gliopathy.” Recent clinical studies have found that people with SCI show synaptic reorganization in the cortex, thalamus, and spinal cord following SCI; they also experience below-level neuropathic pain [155]. However, no studies of SCI in humans have reported the relationship between glial modulation and synaptic reorganization. Therefore, a better understanding of the gliotransmission that leads to persistent neuronal hyperactivity after SCI will potentially aid the development of therapeutic treatment for chronic neuropathic pain.
